# ‘No Man’s Land’: Disability, Rehabilitation, Welfare Policy and the British Ex-Service Migrant in Australia, 1918–39

**DOI:** 10.1093/shm/hkz063

**Published:** 2019-08-03

**Authors:** Michael Robinson

**Affiliations:** History Department, University of Liverpool, 1 Abercromby Square, Liverpool L69 7WZ, UK

**Keywords:** disability, welfare, rehabilitation, veterans, public health, British Empire

## Abstract

An inter-war analysis of the British and Australian departments charged with compensating disabled First World War veterans and the British ex-service migrant in inter-war Australia illustrates how nation-states have failed to unify welfare and disability rehabilitation. Contemporary welfare states continue to codify and establish categories of prioritisation regarding communities with disabilities for public finance administered by national government departments. This binational case study identifies reoccurring type one and type two error problems: policy can deny legitimate claims for state assistance while also validating and financing potentially illegitimate claims. This underlines the factors that dictate which error type is ruled to be the least significant and the impact the resulting model has on individual claimants. This study reinforces the thesis of David Gerber who stresses the ahistorical centrality of ‘biopolitics’ or the relationship between societal and political perceptions of a conflict on state policy, in the treatment of veteran communities.

No research has been published on the disabled British Army veteran of the First World War who resided outside of the British Isles despite the burgeoning literature on the treatment and experiences of disabled ex-servicemen of the British Army.^1^ The oversight of the pensioned ex-service migrant and imperial networks is unsurprising. Few disabled veterans residing in Britain left sources describing their experiences.^2^ Lingering nineteenth-century perceptions of masculinity and the expectation that disabled ex-servicemen should maintain self-control and restrain from articulating their suffering in public influenced this silence.^3^ The shortage of source material increases for disabled British ex-servicemen living abroad. An estimated 23,000 of these men, coined ‘Imperial Pensioners’, were located overseas in 1936.^4^ This figure equated to less than 2 per cent of the overall disabled ex-service population in Britain and Ireland.^5^ Further problems arise with migration scholars attesting to the lack of relevant research resources dedicated to the migrant community in overseas archives.^6^

There is, in contrast, a comprehensive historiography dedicated to British migrants residing overseas.^7^ There has been analysis of the institutionalisation of mentally ill British migrants.^8^ Yet, the physically disabled migrant remains overlooked. To date, only Kent Fedorowich has assessed the experiences of able-bodied British ex-servicemen who migrated to the ‘White Dominions’ of Australia, New Zealand, Canada and South Africa. Fedorowich’s analysis largely focuses on the Overseas Settlement Committee and its associated free passage schemes between 1919 and 1922. These initiatives enabled British ex-servicemen and their families to obtain free third-class boat passages, paid for by the British Government, from their nearest port in the UK to the Dominion or Colony of their choice.^9^

This article provides the first analysis of the disabled British ex-service migrant. Australia is chosen as a case study as it was the most popular overseas destination within the British Empire receiving 16,514 of 37,199 ex-servicemen who settled abroad via the British Government’s Overseas Settlement initiative.^10^ It connects the archival records of the Ministry of Pensions (the British governmental department that provided financial assistance and medical treatment to disabled British ex-servicemen) and those of its Australian equivalent, the Repatriation Commission. These departmental records will be supplemented by the biennial reports of the British Empire Service League (BESL), which was an international federated charity consisting of the leading ex-service charities of each combatant nation within the British Empire.^11^

This article examines the British ex-service migrant whose rehabilitation remained defined by the British Ministry of Pensions but who shared Australian facilities and society with Australian veterans under the remit of the Repatriation Commission. This triangulation highlights and explains the policy differences between the two countries and the subsequent impact on veteran communities and national expenditures. This cross-national framework seeks to highlight the immense historical benefit to the study of veterans through a migratory lens as the existing historiography remains largely constricted to individual nation states.^12^ It will argue that the British Ministry’s programme was hugely undermined without the voluntary assistance of British society.^13^ Regarding Australia, this study enters the historiographical debate on the value of the Commission, siding with Alistair Thomson, Clem Lloyd and Jacquie Rees who have emphasised its liberality as opposed to Kate Blackmore who advances a bleaker view of the Department underlining its austere public economy.^14^ Ultimately, public health, rehabilitation and welfare policy in both countries remains fluid, being shaped by the socio-economic, political and cultural national context in which public policy was devised and implemented.^15^

## The Ministry of Pensions and Repatriation Commission and the Ministry’s First Tour of Australia, 1916–29

Before the First World War, both Britain and Australia provided charity relief funds to returning veterans of colonial conflicts with limited state liability to assist those disabled by war service.^16^ The lethal reality of the industrialised nature of the First World War wounded and disabled mass citizen armies on an unprecedented scale. The Ministry of Pensions was established in 1916 in the UK to provide financial remuneration, employment training and medical treatment to disabled ex-servicemen. The department compensated disabled veterans depending on the severity of their disability. Pensions ranged from 20 per cent awards for the loss of one finger on each hand to 100 per cent pension for the loss of two or more limbs.^17^ The Ministry also provided exclusive rehabilitative and medical treatment for the Great War pensioner. Ministry intervention was at its zenith in 1921 with almost 1,200,000 ex-service pensioners. Segregated hospitals operating throughout Britain and Ireland offered nearly 150,000 veterans specialist in-patient and out-patient treatment for a range of war-related conditions such as limb loss, neurasthenia and orthopaedic surgery.^18^

**Fig. 1.**
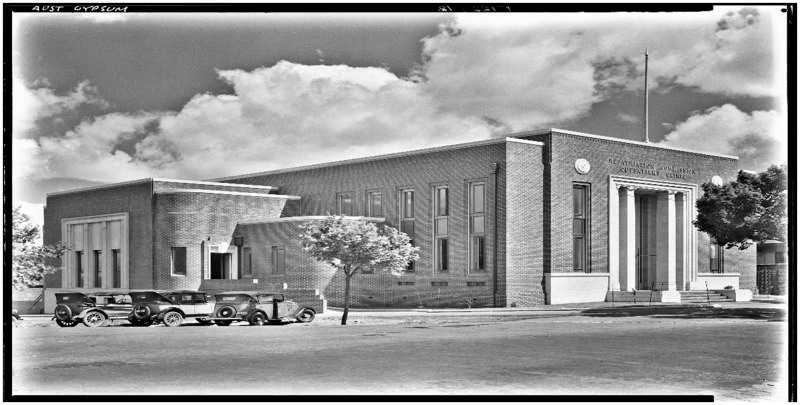
The Repatriation Commission Outpatient Clinic in Melbourne circa 1930–39^21^

Influenced by Britain’s establishment of the Ministry, Australia followed suit with the founding of its Repatriation Commission in 1917.^19^ By 1920, 90,000 veterans of the Australian Imperial Force (AIF) were receiving a war pension across the six states of Australia.^20^ The Government Treasury again funded the Commission, remained bound by statutes and regulations, with a centralised headquarters, regional officials and medical officers working on behalf of the Department across the country. The department also administered medical care to disabled veterans via specialised and exclusive departmental in-patient and out-patient faculties specific to ex-servicemen (Figure 1).

The Australian Repatriation Commission also shared the British Ministry of Pensions’ emphasis on the importance of employment in the rehabilitation of disabled veterans, and of incorporating societal initiatives in the latter.^22^ The two departments were not, however, identical. With provision in Australia being much sparser than in Britain, the Repatriation Commission made more use of associated medical officers interacting with disabled veterans on their behalf.^23^ Reflecting the Repatriation’s emphasis on egalitarianism, the Australian level of disability pension started at a base rate of 5 per cent as opposed to Britain’s preferred 20 per cent. Whereas in Britain the Officer and non-officer class were paid on different scales, Australia had no such class distinctions built into its structure.^24^

In addition to catering for its domestic veteran populations, British and Australian migrants were also rehabilitated and compensated. While the welfare authorities of the migrant’s new host society administered payment and treatment, only their country of origin could approve of new pension awards. The Ministry had a special branch, located at departmental headquarters in London, dedicated to ‘overseas’ cases. The exception here was Canada. Due to the large concentration of around 11,500 pensioners in Canada and the USA, an individual Ministry representative and office was established in Ottawa. The Ministry appeared satisfied with its transnational remit writing: ‘Every effort has been made to ensure that a pensioner abroad has available to him all the medical skill and care of the country in which he is resident’.^25^ Australian authorities also continued to care for their overseas veteran communities. Australia House in London housed Australia’s headquarters for its overseas migrant population. By 1927, there were 9,000 AIF veterans located across the world at an annual cost to the Australian department of over £311,000.^26^

In 1918, Australian Prime Minister Billy Hughes welcomed the participation of British Army veterans in Australian settlements, proclaiming it as a ‘unique opportunity of securing the right type of immigrant’ from British ex-servicemen ‘in the prime of life, who would make most desirable settlers on soil’.^27^ Nevertheless, veterans in receipt of a disability pension were expected to be amongst the ex-service population who migrated via free passage migration as it was publicised in pensioner newspapers.^28^ The British Legion also provided guidelines to pensioners to ensure that their pension was transferred to be paid overseas before their arrival.^29^ Some disabled men, however, were ineligible due to their perceived ailments. Others were able to proceed to Australia regardless of their disability on the assumption that their war wound/s had reached a permanent and manageable status. A third category, noted later in the essay, was made up of those migrants whose disability only became apparent *after* their arrival in Australia.^30^ With regards to those who migrated already classified as disabled, imperial research into immigration screening has a rich secondary literature.^31^ The nature of medical screening of British ex-service migrants to inter-war Australia is, nevertheless, challenging to quantify. Each state had its own medical screening standards, involving hundreds of thousands of applicants, thousands of migration officials and fluctuating state regulations that kept their policy confidential.^32^

Bureaucracy and enquiries regarding suitability were reduced for self-funders. The number of British ex-servicemen who migrated via this method is unknown although Australian officials estimated that the majority of British migrants were self-funders who were not properly vetted before arrival.^33^ While the process of screening ex-service migrants appears convoluted and inconsistent, the arrival of unfit migrants ensured complaints from Australians.^34^ A search of the Australian newspaper archive reveals numerous articles dedicated to British ex-service migrants involved in instances of crime, mental illness and hospitalisation, and letters to the editor complaining as to their costly impact to Australian society.^35^ These concerns seemingly reflected wider concerns that Empire Settlement was Britain’s attempt at ‘pauper shovelling’.^36^

The surge of supposedly unfit ex-servicemen migrating to the Dominions, who subsequently became an alleged burden on Australian society, was cited as a key reason to relieve Australian state officials of this increased liability.^37^ British ex-servicemen residing in Australia were previously cared for via Australian State Treasurers who arranged for the payment and treatment of British veterans.^38^ In early 1921, a Ministry official, Cecil Floersheim, visited Australia to report on transferring administrative responsibility to Australian Repatriation authorities. Both Floersheim and Repatriation officials recommended a transfer citing the long delays and confusion regarding correspondence between officials in London and Australia.^39^ P. F. Aitken, the Ministry official who headed the administration of ‘overseas’ cases from London, advocated passing *complete* jurisdiction over the British ex-service migrant to the Repatriation.^40^ Ian Macpherson MP, the British Government’s Minister of Pensions, dismissed Aitken’s suggestion believing that British pensioners should be treated equally regardless of location. Tellingly, Macpherson also held the Australian administration to be too liberal and costly. Therefore, the British Treasury would have strong objections to providing Australian authorities with the unsupervised power to distribute its capital.^41^ This stance was agreed upon by Adair Hore, one of the leading officials within the Ministry, who told Aitken that the Ministry had to avoid giving ‘carte blanche’ to Australian authorities in distributing Ministry funding.^42^ Hore maintained that the Treasury would never agree to pass complete control to the Commission as it was impossible to provide accurate estimates as to future costs.^43^ The process of the Commission acting as an agent on behalf of the Ministry and vice versa was nevertheless completed by 1923.^44^

Hore recognised that Repatriation officials believed the Ministry’s rules to be ‘excessively restrictive’.^45^ Summarising their supposed fundamental differences, A. Skerman, both a Repatriation official and its official historian, held that Australia accepted all financial and legal responsibility for its disabled ex-servicemen regardless of the existing economic context. In contrast, the national economy and the availability of finances shaped British policy. It was within this austere outlook, he argued, that the Ministry placed a large emphasis on social bonds and philanthropic bodies to help reintegrate British veterans back into the society.^46^ One prominent scheme was the King’s National Roll, a state programme that encouraged employers to ensure at least 5 per cent of their workforce were ex-servicemen in receipt of a disability pension. Launched in Britain in September 1919, employers who participated were included on a national ‘Roll of Honour’ able to use the King’s Seal in correspondence. Previous research into the scheme highlights its worthiness as almost 24,000 employers participated in helping 259,000 disabled ex-servicemen attain employment in 1921. Such an effort helped to create a national sense of duty and appreciation to the disabled ex-service population.^47^

There was an increased potency of ex-service lobbyists and in the political capital associated with veteran issues in Australia.^48^ Researchers have attributed the increased liberalisation of the Repatriation Commission to the ‘Anzac Legend’, which was a widespread perception that the Australian Imperial Force’s sacrifices during the Great War gave birth to the nation of Australia.^49^ The Returned Sailors and Soldiers Imperial League of Australia (RSSILA) became an ‘effective champion’ on behalf of the Australian veteran due to its lobbying, campaigning and personal assistance in furnishing claims and employment by repeatedly using notions of the ‘Anzac Legend’ to its advantage.^50^

Differences between the British and Australian schemes became further magnified with the UK’s implementation of the ‘Geddes’ Axe’, which enforced a host of tax increases and economic cutbacks across the UK public sector from 1921 onwards.^51^ The cutbacks were a primary reason for the restriction of government assistance in Empire Settlement.^52^ The British Treasury wrote to all pensions departments urging austere economic policies throughout the department. The War Pensions Act of 1921 ushered in a subsequent period of restrictions to the Ministry’s policy and procedure in Britain. Overall spending for the department was reduced by almost 50 per cent from £106,367,000 in 1921 to £54,066,000 in 1930. This reduction was achieved in numerous ways. By 1925, almost half a million pensioners, with around 50 per cent of those in receipt of a pension scaled at 20 per cent or less, were given a lump sum or gratuity defined as a ‘Final Award’. There was a subsequent reduction in medical facilities: while 332,000 disabled pensioners were undergoing treatment in 1921, this figure stood at just 41,000 by 1930. The Ministry then often covered the cost of a veteran’s health care via capitation grants for treatment in public hospitals or via their personal General Practitioners.^53^ A subsequent associated reduction in Ministry staff resulted in 21,685 departmental staff in 1921 being reduced to just 3,795 a decade later.^54^

This reduction in Ministry liability and state intervention was seemingly aided by the lessening in societal concern for the disabled First World War veteran as the British population became desensitised to their plight in a time of widespread austerity and employment scarcity.^55^ The Ministry’s 1921 legislation further restricted the potential for future state liability by introducing a 7-year time limit on claims from discharge or the end of the war.^56^ This position was not mere draconian penny-pinching. Both the BESL and even the Ex-Service Welfare Society, an otherwise staunch advocate on behalf of the mentally ill veteran in Britain, conceded the difficulty in tracing the relationship between war service and ill health over more than a decade.^57^ The British Treasury was fearful of being burdened by the financial consequences of non-war-related ill health or the burden of ageing veterans.^58^ The Ministry, Parliament and Ministry of National Service were acutely aware of the apparent abuses within the American Civil War pensions system.^59^ The liberal American system became, to all intents and purposes, an old-age pension. By 1914, 93 per cent of Union veterans, nearly 400,000 men, received a state pension for a war-related disability.^60^

The Australian department saw things differently citing the differences in ‘basic principles governing the provision’ of the Australian and British systems:

It might be said that the Australian ex-soldier is offered every facility to establish his claim for the necessary facility to establish his claim for the necessary treatment, whereas the British man has to prove his right to treatment and is not helped to the same extent by the Ministry.^61^

These contrasting standards of principle, in the opinion of one Repatriation official, explained why the relative cost of pensioners receiving treatment in Australia was much higher than the UK.^62^ The comparative generosity of the Commission to the Ministry was also repeated in the Australian House of Representatives.^63^ J. M. Shemmens, Chairman of the Repatriation Commission, criticised his British counterparts and their ‘definite steps to limit its post-war expenditure’, writing: ‘the British Ministry is definitely illiberal’, paraphrasing the Ministry’s alleged mission as ‘getting the patient off our hands as soon as possible’. Shemmens praised the Commission’s treatment of Australian veterans, which had been backed by successive federal governments, concluding: ‘the comparison between the two administrations may be reduced to this: the Repatriation Commission “cares” for its patients the British Ministry of Pensions does not’.^64^

Such simplistic and moralistic conclusions are complicated, however, by considering the resulting impact that Australian welfare legislation had on national expenditure. The Commission and its Chairman, for example, accepted that it was impossible for its associated medico-pensions official to decipher whether a fresh disability claim made years after discharge was war-induced.^65^ The Commission noted the unexpected peak of fresh claims in 1927 where the chances of success still appeared stacked in the Australian veteran claimants’ favour, with just 2,518 rejections out of 13,323 new claims. Reviewing the year, the department conceded that the ‘exceedingly liberal’ remit of its welfare policy, shaped by the ‘expressed desire’ of successive governments to make sure veteran welfare was ‘interpreted in the most generous manner’, explained the subsequent acceptance of state liability. The absence of any time limit was critical in the increased claims for state assistance. In contrast, the Commission pinpointed the British Ministry’s 7-year time limit as the fundamental factor in its comparatively reduced financial outlay the same year.^66^ The BESL protested against the Ministry’s 7-year time limit on British claims believing it to be prejudicial against its ex-servicemen in acquiring fair recompense for war-related disability.^67^ It was within this context that the Ministry’s first tour of Imperial Pensioners in Australia occurred. An analysis of the department’s resulting report reveals the nuanced situation regarding state responses to post-conflict disability and rehabilitation.

Between 1925 and 1926, following an audit of administration carried out on behalf of the Ministry in North America, G. F. Gilbert, a senior official in the Ministry’s accounting department in London, visited India, imperial territories in British Africa, Australia and New Zealand.^68^ In Australia, Gilbert met associated Repatriation officials, Medical Advisory Committees, Departmental Medical and Pension Officers, the Managers of Artificial Limb Factories and members of various ex-service associations. Gilbert also visited four Repatriation Hospitals, four exclusive Sanatoriums, one state mental institution and two Anzac Hospitals.^69^ As is often the case with disabled communities, the majority of Imperial Pensioners lived *outside* of medical institutions.^70^ Most Imperial Pensioner cases appear to have been of disease and illness such as bronchitis, tuberculosis, heart problems and psychoneurotic ailments (Table 1). The provision of treatment generally constituted Imperial Pensioners attending a Repatriation Commission out-patient clinic twice a month for a supply of alleviating medicines.^71^ Gilbert estimated that there were 651 Imperial pensioners receiving treatment. Just over a third were receiving in-patient treatment, half via out-patient care and 15 per cent via associated medical officers:

**Table 1.** Medical treatment and number of Imperial Pensioners, 1925^72^

Disability	Number of Imperial Pensioners
Tuberculosis	130
Bronchitis and asthma	98
Neurasthenia	98
Insanity	65
Rheumatism	26
Malaria	20
Misc. other	182

As an auditor, Gilbert’s primary function was to emphasise Ministry instructions and conditions to Repatriation officials. In total, the annual outlay paid for by the Ministry amounted to £255,000.^73^ Gilbert reserved praise for the Branch Offices in New South Wales and Queensland, with issues involving Imperial Pensioners devolved to one or two staff members.^74^ Gilbert, however, also noted a widespread lack of coordination of understanding between Repatriation Branches and Ministry officials, which came at a financial cost to the Ministry. Gilbert concluded that his explanation of Ministry regulations would enable a better understanding of British policy and account for a reduction totalling £1,000 per year.^75^ Gilbert denied any venture that would allow the Repatriation Commission to incorporate and categorise Imperial Pensioners as disabled Australian veterans with the Ministry covering the bill.^76^ Gilbert’s report also devoted considerable attention to the functioning and remit of Repatriation staff infrastructure. These observations underline the contrasting remit and functions of British and Australian infrastructures.

Gilbert would also recognise the comparative liberality of the Commission. When discussing Repatriation medico-pensions officials examining veterans suffering from tuberculosis, he wrote that they largely attributed their condition to war service with little concern if it was induced by active service: “The Committee certainly give the benefit of the doubt to the applicant. In fact, I do not think it would be unjust to them to say they generally spend some time looking for the ‘shadow of doubt”. Gilbert referenced further legislative differences between Ministry and Repatriation policy, including the aforementioned lack of time limits and final awards.^77^ Gilbert cited the Australian socio-political context to explain the existing benevolence:

This liberality was born of the spontaneous sympathy and generous impulses of the Australian people, but I am afraid that it is now kept alive and fostered by the exigencies of politics. The political element apparently enters far more into pension matters in Australia than in Great Britain.^78^

Further distinctive political infrastructure in Australia improved the ex-serviceman’s position. While there was a Federal Parliament for the whole of Australia, each state had its own Parliament. This increased regionalism and democratisation gave ex-service bodies more political leverage:

Every member of the Parliament deemed it his duty to obtain as much as possible for his constituents, irrespective of whether they were entitled to it or not. There is no doubt, also, that the Associations of ex-servicemen are able to exert far more pressure in the Commonwealth than in Great Britain.^79^

Gilbert interacted with a small number of Imperial Pensioners receiving in-patient treatment at Repatriation facilities. They declared their contentedness and comfort in the facilities provided. Included within Repatriation medical infrastructure, for example, were sanatoriums and mental hospitals reserved for tubercular and insane ex-servicemen, respectively. Such segregation of insane ex-servicemen supposedly spared them from the stigma of hereditary and degenerative mental illness.^80^ On visiting one such facility in Victoria, which housed three Imperial Pensioners, Gilbert noted the ‘lavish provision made for the comforts of the patients, the appointments being really suggestive of a first class hotel’.^81^ By comparison, ex-servicemen in Britain suffering from tuberculosis and insanity shared public facilities with the wider population. With regard to the latter, previous research has foregrounded the austere and highly stigmatised asylums that became associated with Great War veterans.^82^

Australian treatment did not always surpass comparative treatment available in Britain. The British Legion, medico-military staff and contemporary historians have assessed that the British metal leg limb was superior regarding durability and flexibility in comparison to the wooden limb favoured in Australia.^83^ On visiting five artificial limb manufacturing sites across Australia, Gilbert wrote that the artificial limbs constructed and distributed were of an inferior wooden variety. While the number of limbless Imperial Pensioners in Australia was unknown, 12 were provided with Australian-made limbs in the year before Gilbert’s tour.^84^ Writing about the Imperial Pensioner overseas, the Ministry held ‘every effort has been made to ensure a pensioner abroad has available to him all the medical skill and care of the country in which he is resident’.^85^ A case study of the Imperial Pensioner in Australia and their allocation of in-patient facilities and artificial limbs thus highlights the dangers of offering a universal illustration of national responses to disability. The treatment of a disabled British veteran depended on their diagnosis. While the insane Imperial Pensioner received superior treatment in Australia compared to their similarly afflicted former comrade who remained in Britain, the opposite appears true regarding those with artificial legs.

The crucial role of the family and philanthropic lobbyist also needs to be highlighted. Even when Australian in-patient facilities were superior to what was otherwise available in Britain, British ex-service migrants did not always benefit from these because of the absence of a family member or an ex-service association submitting a claim to the Ministry on their behalf. Some British ex-service migrants were treated in highly stigmatised and overcrowded public asylums overseas as pauper lunatics, with no recognition of their former war service.^86^ Further national policy divergences arose between the British Ministry of Pensions and the Australian Repatriation Commission in the 1930s, which increased discontent amongst the British ex-service migrants residing in Australia.

## Increased National Policy Divergence and the Ministry’s Second Tour, 1930–39

In contrast to the Ministry’s extensive reduction of exclusive medical facilities, funding for veteran medical facilities continued in Australia into the late 1930s, including extensive renovation in comforts and medical equipment throughout the country and the establishment of modernised out-patient facilities.^87^ Australian wooden prosthetics, however, continued to be inferior to the metal devices available to ex-servicemen who remained in Britain.^88^ Thomson emphasises that the strength of the ‘Anzac Legend’, and the special national categorisation of the Australian veteran, returned to the fore in improving welfare policy regarding AIF veterans during the 1930s.^89^ Seemingly taking the lead from Canada, and having been previously discussed and promoted by the BESL, the Repatriation Commission responded to public and political lobbying on behalf of the Australian veteran to introduce the so-called ‘Burnt Out Digger’ legislation.^90^ This measure aimed to facilitate Australian veterans who had hitherto not applied or had been rejected for relief or assistance. Repatriation concessions included providing a pension to previously uncompensated veterans who reached the age of 60 years, as well as making eligible those who had proven to be permanently unemployable and easing the success rate of tubercular claims. The latter was particularly aimed at those servicemen who had been gassed during the war, and whose disability was believed to have been due to the deleterious and long-term impact of war service on reducing a veteran’s immune system, ensuring their supposed susceptibility to contracting diseases such as tuberculosis. From 1936, the Commission allowed any tubercular ex-servicemen to receive a pension and medical treatment regardless of the war’s perceived attributability.^91^

The British Treasury and Ministry, in contrast, rejected identical lobbying on behalf of what the British termed as the ‘prematurely-aged ex-serviceman’ due to its 7-year time limit on claims. Despite the British Legion persistently agitating for the removal of the time limit, which included a deputation with the Minister of Pensions F. O. Roberts in June 1927, and associated medical conferences and articles, new claims were very rarely accepted in the late 1920s and 1930s.^92^ Repeated questions from MPs to the Minister of Pensions in the House of Commons were similarly rebuffed.^93^ The perceived draconian nature of the time limit led the British Legion in 1933 to describe the Ministry as using ‘medieval methods’ in its dealings with claimant ex-servicemen.^94^ The Ministry countered that its limited time response to progressive ailments was a necessary ‘cornerstone’ of British policy:

Evidence has always, therefore, been essential … in the reliance of this evidence, the Ministry have been able to resist the occasional demand that a definite presumption of war should be embodied in Warrant or legislation.

Tellingly, the Ministry cited that the Repatriation Commission ‘have to their disadvantage, in the absence of precisely this evidence, been driven to adopt’.^95^ The Ministry went so far as to argue that the awarding of maximum pensions for tuberculosis in the Australian scheme was actually ‘prejudiced’ against the state and the public purse.^96^

Repatriation concessions had their detractors in both Britain and Australia. Writing to the Auditor General of the Melbourne state government, Gilbert wrote in 1934: ‘I certainly share your views that there is too much economic waste and extravagance in the commonwealth’.^97^ The political potency of the Australian veteran bloc was again cited as the fundamental explanation to such concessions:

As you say, the members of the Commonwealth parliament seem to be more keen on catching votes than on conserving the resources of the country, and the government certainly gives way to them much more readily than our government does.^98^

Historians who have analysed the efforts of the Repatriation Commission also suggest that the department may have underestimated the potential for abuse its liberal infrastructure allowed.^99^ Gilbert Dyett, Dominion President of the BESL, nevertheless, pressed Britain to follow the example of Australia and Canada and introduce equivalent legislation to assist fresh claims at a BESL conference in 1937.^100^ By way of a response, Harwald Ramsbotham MP, the Minister of Pensions, stated: ‘The British pensions system was, on the whole, the best in the world’. Ramsbotham justified the efforts of successive British governments to keep pensions out of politics and to deny the Ministry from becoming entangled with wider social unemployment and public ill health, which ensured the efficiency of the British system. Ramsbotham felt such measures were increasingly necessary during a period of high financial debt.^101^ At a BESL conference, 3 years earlier, Sir Ian Fraser MP similarly advocated for the prioritisation of the national economy arguing: ‘ex-servicemen could not prosper unless their country prospered’.^102^

**Fig. 2.**
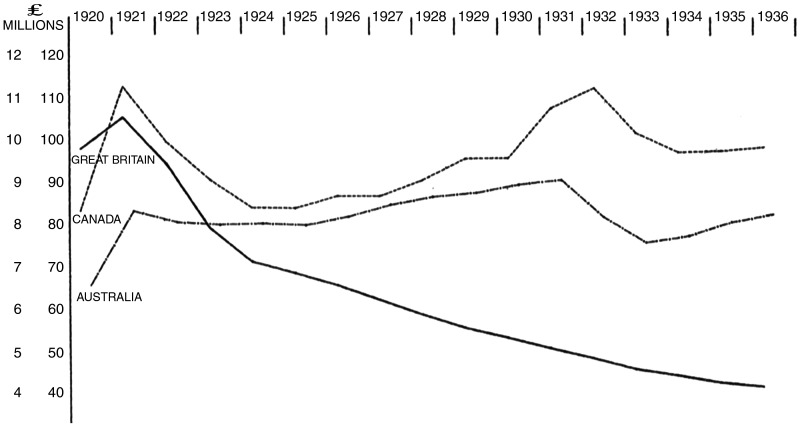
Annual expenditure of Britain, Australia and Canada for pensions and allied medical services, 1920–36^104^

These contrasting welfare models had significant economic repercussions. While the financial outlay of the Ministry had been reduced by almost 51 per cent between 1921 and 1930, Repatriation costs increased by 25 per cent during the same period.^103^ A simultaneous financial trajectory of the Ministry and Commission highlights the former's reduction in costs juxtaposed with Australia's increase in expenditure (Figure 2). Around 43 per cent of Australian veterans had applied to the Commission for a pension by 1937.^105^ By the late 1930s, Repatriation costs constituted one-fifth of the Australian Federal Government’s expenditure, becoming the largest single expenditure within Australian welfare or social services.^106^ This incremental growth of Repatriation policy remit, function and economic cost would be defined by researchers of the contemporary welfare state as welfare ‘creep’.^107^ In contrast, by 1930, and with the department’s financial outlay lessening by the year, Ministry spending totalled less than 5 per cent of British Government spending.^108^ It is, of course, necessary to move beyond simplified annual expenditures to fully understand differences in national welfare policy and infrastructure. An analysis of the Ministry’s second tour of Australia in 1935/36 effectively illustrates the fundamental policy differences between the two counties and their impact on veterans.

The Ministry’s second inspection of Australia, lasting for 8 months between 1935 and 1936, was undertaken by W. F. N. Smith.^109^ Smith estimated that he dealt with 300 individual cases involving British claimants interviewing around 130.^110^ Previously rejecting requests to set up a Ministry branch in Australia akin to the office located in Ottawa, Canada, Ministry officials believed the ‘pill would be gilded’ if they conceded to an official presence.^111^ Like Gilbert, Smith assessed that the remit and functioning of the Commission fluctuated by state.^112^ Ministry regulations were seemingly not understood by Imperial Pensioners, Repatriation officials or ex-service charity workers in Victoria, Tasmania, South Australia and Western Australia. Repatriation authorities and Imperial Pensioners were even unaware of the right to appeal a ‘Final Award’ that had lapsed unless substantial new medical evidence had arisen or were aware that the Ministry had a 7-year time limit on claims.^113^

Smith heard repeated criticisms of the treatment of British migrants throughout his tour by Commission officials, charity workers and British migrants alike. Smith reasoned ‘the Imperial in Australia, without effective local representation from the Ministry, felt himself to be neglected by his own government and treated with a lack of sympathy and inadequate knowledge by the country of his adoption’.^114^ Smith countered during numerous personal meetings with Repatriation officials that any problems were the result of a ‘lack of authoritative information rather than any serious defects in the system’.^115^ There was a widespread feeling amongst Britons that it was impossible ‘to get beyond the agents’ and communicate directly with the Ministry who were unable, rather than unwilling, to intervene.^116^ One British migrant, who had unsuccessfully submitted a pension claim through the Repatriation Commission, told Smith: ‘Frankly I do not believe the Ministry of Pensions gave such a decision’. The migrant sardonically joked that his claim was cast aside by the ‘Repudiation Department’ without reference to the Ministry.^117^ Ignorance of British regulations regarding time limits ensured that large amounts of medical documentation submitted to the Ministry on behalf of the British ex-service migrant were often incomplete, incorrect and ineligible. The unverifiable nature of many claims, requests and forwarded documentation ensured that many were ‘often utterly useless’ and destined for rejection.^118^ Smith thus held that the establishment of a departmental branch in Australia would only serve to give false hope to British migrants whose ineligible and inadequate claims for relief and rehabilitation were fated for failure.^119^

Migrant ex-servicemen in Australia were further hampered by their isolation. Empathetic judgements appear increasingly unlikely if claims were based only on paperwork as opposed to personal testimony.^120^ British settlers in Australia were disadvantaged in this regard as they could only personally attend a hearing if they financed and arranged their return to Britain.^121^ In his analysis of veterans of twentieth-century conflicts, Niel Diamant comments that ex-servicemen fared better if they were able to organise autonomously or semi-autonomously.^122^ Unlike their former ex-comrades who remained in Britain, British migrants did not benefit equally from the support of the British Legion who assisted veterans in Britain with employment schemes, charitable donations, training opportunities, employment, pension claims, and information on issues such as limb fitting.^123^ Even when Australian ex-service associations endeavoured to assist British ex-service migrants, officials and charity workers did not have access to the personal files of claimants.^124^ There was, in contrast, an opportunity for Australian veterans residing in Britain to have their case heard in person by Australian officials at Australia House in London.^125^

Even with valid claims, the distance from Ministry officials in London caused substantial delay in communication and gaining approval. A period of 12 months was the average for an official decision on a claimant’s response.^126^ The laborious administration could delay much needed medical treatment. One Imperial Pensioner was receiving emergency medical treatment at a Repatriation Hospital in Victoria for a gunshot wound. Still awaiting a decision from the Ministry, he was forced to finance his in-patient treatment personally.^127^ Recognising these lengthy delays, the Ministry accepted Smith’s two primary recommendations. Firstly, for the increased use of airmail when transferring correspondence between Australia and Ministry headquarters in London. Secondly, they agreed to the increased distribution of the British Legion’s *Great War Pensions* (1924) booklet to provide clear and concise information regarding Ministry policy.^128^

A council of ex-service charity workers in Melbourne held that most of the dissatisfied applicants amongst the British ex-service migrant population were those who had sought assistance only when their health had broken down *after* their arrival in Australia.^129^ Smith similarly conceded: ‘In so many cases there has been consequent distress despite a heroic struggle, during which health may have been failing’.^130^ One constant feature in disability and welfare studies has been the apparent correlation between unemployment, poverty and claims for relief.^131^ In post-war Britain, medico-pensions officials repeatedly attested to the correlation between unemployment and subsequent claims for pensions amongst psychoneurotic pensioners.^132^ AIF ex-servicemen’s claims for relief similarly increased during the Depression of the 1930s with unemployment widespread in Australian society.^133^ This relationship becomes apparent in a study of the British ex-service migrant in inter-war Australia.

Disability and relief claims were, for example, evident amongst struggling British migrants who attempted to settle in the mining districts of New South Wales.^134^ Repatriation officials in Perth and ex-service charities in Brisbane also relayed to Smith that the majority of Ministry claims were fresh appeals from destitute British ex-servicemen during the widespread economic depression that affected the Australian economy in the 1930s.^135^ Oral testimonies of Australian men and women recounting their experiences of living in Australia during the economically depressed 1930s cite personal anecdotes of ex-servicemen’s mental state being worsened by their unemployment, even driving some to commit suicide.^136^

British settlers shared the fate of Australian ex-servicemen whose health was detrimentally affected by working on failing land settlements.^137^ Smith visited some settlements in Western Australia and described the depressing conditions experienced by British settlers: ‘It required no expert eye to realise that some of the propositions offered were, to say the least, impracticable; and the conditions under which some of the settlers were still living were terribly primitive’.^138^ In November 1934, breaking off from its conference in Melbourne, BESL delegates also toured nearby land settlements shared by British and Australian ex-servicemen. Describing the dire and isolated conditions of the remaining farms, the charity vowed to provide more help to those involved: ‘The world was beginning to forget the ex-serviceman, but they had a duty to the living as well as to the dead’.^139^ Oral testimonies provided in the early 1990s by the children of British ex-servicemen who attempted land settlement similarly attest to the desperate and isolated conditions of migrants and their families during the depression, and the increased anxiety and mental strain induced on them as a result of these unfortunate circumstances.^140^

Writing to the House of Commons in Britain, one newly formed association of British ex-service settlers in Victoria pleaded for British governmental assistance. Describing the settlement schemes as ‘a hollow sham’, many British ex-servicemen were claimed to be ‘in a state of dire poverty and want … Settlers soon abandoned holdings to avoid starvation, while departments looked on and did nothing’. Both the Secretary of State for Dominion Affairs and the Commonwealth Treasurer proved unwilling to intervene on behalf of the British migrant responding that any complaints were to be addressed to the relevant Australian authorities.^141^ The British Government’s non-intervention to relieve these communities echoed wider Imperial policy with the British Government repeatedly reluctant to provide any form of after-care for migrants in Australia.^142^ In comparison to the disabled Australian veteran, the RSSILA also seemingly provided little in the way of intervention on behalf of the veteran British migrant who settled on the land.^143^

The dire and isolated position which many ex-service migrants found themselves is highlighted by the deaths of ex-servicemen who died in destitute circumstances and were buried in pauper graves. Unless the migrant’s death was related to a pensionable ailment, the Ministry proved unwilling to cover funeral and burial expenses, sparing the deceased veteran this shame.^144^ Australia, in contrast, used public donations for its ‘Last Post’ scheme, which financed the burial of ex-servicemen regardless of their welfare status.^145^ While no overall national numbers are cited, the RSSILA in Western Australia estimated around 50 such instances would arise in the state involving British veterans during the year of Smith’s visit, articulating their indignation at the Ministry’s inactivity. Smith had sympathy for this outrage while holding little hope the Ministry would adjust its position.^146^

Previous research has described the Australian inter-war infrastructure as being ‘truly exceptional’ in its treatment of its ex-servicemen, even benefiting those who had not suffered considerably due to their war service and those affluent enough to be financially secure without additional government entitlements. This ‘welfare apartheid’ would ensure sporadic resentment aimed at ex-servicemen from the non-veteran communities.^147^ The British migrant seemingly shared this discontent with many non-veteran contemporaries in their host society.

Smith conceded the administration of rehabilitation by the Ministry in Australia was not as ‘all embracing’ as that which the Commission afforded its ex-service population.^148^ Smith recognised that comparisons were inevitable and could magnify the British ex-serviceman’s sense of injustice.^149^ The significant number of British-born veterans of the AIF who received superior treatment under Australian legislation only served to heighten the British migrant’s sense of injustice.^150^ The cost of living differences between Britain and Australia intensified bitterness. In a letter to his local Repatriation Branch, Imperial Pensioner, P. E. Causwick articulated his resentment at the reduced purchasing power of British pensions for those living in Australia. Seemingly struggling to afford necessary items such as food, drink and rent, Causwick complained that ‘the question of subsequent migration of ex-servicemen to Australia was not allowed for’.^151^ This negative connotation of comparison was agreed upon by Repatriation official, W. Keays, who, in August 1935, wrote: ‘As regards complaints from British ex-servicemen, it is the opinion the majority received—whether oral or written—can be attributed to a comparison of the two policies, vis, British and Australian for the payment of war pensions and the provision of medical treatment’.^152^ Repatriation Officials in Melbourne went onto explain:

It seems only natural that more complaints proportionally will be received from British pensioners residing in Australia, than those resident in the UK, as the British pensioner will come in contact nearly everywhere with the Australian war pensioner, who receives a more liberal deal so far as pension matters are concerned.^153^

Smith further advanced that the relative liberalism of the Repatriation Commission would inevitably give rise to discontent amongst British settlers:

It is not easy in such an atmosphere to discuss freely the hard facts which must on occasion override sentiment. All these factors, and others of similar nature in the Australian scheme, inevitably tend to create dissatisfaction with the administration of a system which, by the test of time, has proved adequately to meet conditions in the UK.^154^

Australia’s distinct socio-political circumstances continued to influence the increasing divergence in national welfare policy. As Smith attested:

Ex-service questions [were] always a live issue in Australia … political considerations provide an active part in pensions policy, and in Parliament by constant representation in the press and on wireless programmes, the public is continuously reminded of the demands or aspirations of the ‘diggers’.^155^

Claims for welfare or medical treatment were hampered by Ministry regulations, which stressed continuity of medical evidence directly related to war service in association with its 7-year time limit.^156^ As Repatriation officials noted, due to this time limit on British claims, it was a standard procedure for the Ministry to reject claims if the claimant was not already officially classified on their pension roll.^157^ The Medical Superintendent at a Repatriation Hospital in Brisbane told Smith that Repatriation medico-pensions officials dealing with British claims believed Ministry time limits and ‘Final Awards’ to be ‘rigidly harsh’ and ‘dishonest’. Repatriation officials in Perth headquarters similarly held that Ministry regulations were ‘underhand’.^158^ Another senior Medical Officer of the Commission felt the Ministry were unfair in placing a time limit on claims. He believed the majority of claimants were hard-working and honest men who had resolutely persevered with their medical conditions before eventually applying for help.^159^ Smith was told of seven recently rejected British claims in Melbourne of which at least five would have been accepted under Australian legislation.^160^ This instance highlights both the comparative liberality of the Australian system, as well as the previously cited financial incentive to not pass on the jurisdiction of the Imperial Pensioner to the Commission.

In Brisbane, RSSILA officials held the lesser pensions provided to tubercular Imperial Pensioners were unjust and miserly.^161^ On visiting a Repatriation sanatorium in Victoria, which was shared by both AIF and British veterans, one Imperial patient asked Smith if his pension could be increased to the maximum 100 per cent allowance in line with the recent recompense provided to his fellow Australian patients. On receiving a negative reply, and in full-view of other patients, the patient coldly dismissed Smith and returned to his game of cribbage.^162^

The plight of the British ex-service migrant in Australia was also reflected in their social standing. Not only was the King’s National Roll not extended to Australia, but also British ex-service migrants suffered as a result of a lack of inclusion and protection within Australian Federal law, which decreed that government positions must be filled by certain quotas of Australian ex-servicemen.^163^ British veterans were even discharged from their positions to accommodate the Australian veteran.^164^ Writing to the Premier of the Tasmanian Federal Government, one British migrant complained that the prioritisation of Australian ex-servicemen ensured that ‘it is hopeless for an ex-imperial soldier’ to attain employment.^165^ On hearing complaints of the preference of Australian veterans to British ex-service migrants at the seventh biennial conference of the BESL, W. Kemp, an official for the Australia ex-service charity, the Old Contemptibles, told delegates that such measures were ‘quite right’ and ‘common sense’, attesting:

If any promises were made, they had been made to men of the AIF who went away on service. This preference was now constituted as one of the methods of Repatriation. If anybody was responsible for the repatriation of British ex-servicemen that was the British government not Australia. If the government of Australia agreed to the extension of the principle of preference to Ex-Imperial Service Men, there would probably by an influx of them into Australia right away, and Australia’s own men would be out of work.^166^

Smith similarly noted a ‘definite antipathy on the part of the Australian public to providing for Imperial ex-servicemen … the general attitude was that if the disability were connected with the war, the responsibility should be that of the Imperial Government’.^167^ Like the wider British migrant population who settled in inter-war Australia, the story of the British First World War veteran who migrated to Australia often appears to have been an unhappy one.^168^ Smith similarly concluded that Australia was ‘a hard home for the distressed especially to those from other countries’.^169^

The Ministry’s policy and procedure were thus judged to be inadequate amongst ex-service bodies in Australia.^170^ A Repatriation official in Canberra assumed that there would be a continuation of the isolation of the British ex-service migrant who ‘feels neglected by his own people, and not sympathetically treated by his adopted country’.^171^ Psychologically, sharing a country with inadequate rehabilitation infrastructure with a more revered veteran community appears to have worsened the situation. The Secretary of the TB Sailors and Soldiers Association in Victoria held ‘there was an attitude of futility on the part of the men concerned, and this was detrimental to their health’.^172^

By the summer of 1939, just months before the outbreak of a second global conflict, the Commission was still describing the ‘considerable numbers’ of fresh claims from AIF veterans.^173^ In 1933, eight years after visiting Australia, Gilbert wrote to the contrary that the Ministry was ‘gradually dying and, unless we have another war, a few years should see ‘RIP’ written over its portals’.^174^ The Second World War would indeed confirm the Ministry’s survival. The mass migration of Britons, which included ex-servicemen, to Australia in the immediate years following the second global conflict would ensure the eventual establishment of an official Ministry departmental branch in Adelaide. Following Gilbert and Smith, the Ministry’s next tour of Australia occurred in 1952 considering the treatment of ex-service migrants of *both* the World Wars.^175^

## Conclusion

The Ministry wrote in 1934 that no government department had been criticised more across British society.^176^ Four years earlier, the Commission summarised the difficulty facing nation-states and their rehabilitation of disabled the First World War veterans: ageing and associated impairments of health, and the difficulty in objectively assessing the attributability of war service.^177^ The BESL recognised that government departments across the British Empire faced immense difficulty in their tasks and that it was not fair to criticise them too harshly.^178^ It remains difficult to judge objectively which national route was superior. At their most basic levels, the Australian system appears to have benefited individual veteran claimants, whereas the British remit protected public expenditure. This article complicates previous assertions that Britain and Australia treated its returning veterans in generally similar ways.^179^ An inter-war analysis of the Ministry of Pensions, the Repatriation Commission and the British ex-service migrant instead illustrate how modernity has failed to achieve uniformity and satisfaction amongst western nations and their distribution of welfare and rehabilitation.^180^

Contemporary welfare states continue to establish categories of prioritisation for public finance administered by national government departments. This binational case study identifies reoccurring type one and type two error problems in the administration of disability-induced welfare: policy can deny legitimate claims for state assistance while also validating and financing potentially illegitimate claims. Previous historians of rehabilitative departments have cited that pensions and treatment became a battleground in which a ‘protracted civil war was fought’ between veterans, families and charities versus state apparatus.^181^ In Britain, the state’s increased austerity and integration of social responsibility amongst its citizens in their welfare and rehabilitation programmes were fundamental. In contrast, in Australia, ‘the battle for the Anzac legend’ was won by those who regularly foregrounded the sacrifices of Australian veterans who featured prominently in national and public commemorations throughout the inter-war period for having given rise to the birth of Australia.^182^ This article reinforces the thesis of David Gerber, an authoritative voice in the history of disabled veterans. Gerber correctly cites the seemingly ahistorical centrality of ‘biopolitics’, the relationship between societal and political perceptions of a conflict on state policy, in the analysis of the reception of returning veteran communities.^183^

Ultimately, the British ex-service migrant did not benefit from British societal assistance integral to the Ministry’s rehabilitation of disabled veterans. To magnify their discontent, those residing in Australia shared a country offering more generous and liberal compensation packages to its disabled veterans, where AIF-veteran rights were ring-fenced by a sympathetic public who not only protected but also *improved* their legal and political rights. With regards to the British ex-service migrants who shared Australia with AIF veterans, and to borrow from the lexicon of the First World War, they were positioned in a ‘no man’s land’. 

## Footnotes

1 Deborah Cohen, *The War Come Home: Disabled Veterans in Britain and Germany, 1914*–*1939* (Berkeley: University of California Press, 2001); Peter Barham, *Forgotten Lunatics of the Great War* (New Haven: Yale University Press, 2004); Fiona Reid, *Broken Men: Shell Shock, Treatment and Recovery in Britain, 1914*–*1930* (London: Continuum, 2010).

2 Meaghan Kowalsky, ‘Enabling the Great War: Ex-Servicemen, the Mixed Economy of Welfare and the Social Construction of Disability, 1899–1930’ (PhD thesis, University of Leeds, 2007), 48.

3 Cohen, *The War Come Home*, 130.

4 Nineteenth Annual Report of the Ministry of Pensions [hereafter MoP], 1935–1936, Part Two, 5.

5
*Ibid.*, Part One, 14.

6 Eric Richards, *Destination Australia: Migration to Australia since 1901* (Sydney: University of New South Wales Press, 2008), 10; Michael Roe, *Australia, Britain, and Migration, 1915*–*1940: A study of desperate hopes* (Cambridge: Cambridge University Press, 1995), 254.

7 This historiography is extensive. For an introductory study of the inter-war period, see Stephen Constantine, *Emigrants and Empire: British Settlement in the Dominions between the Wars* (Manchester: Manchester University Press, 1990).

8 For example, see Catharine Coleborne, *Insanity, Identity and Empire: Immigrants and Institutional Confinement in Australia and New Zealand, 1873*–*1910* (Manchester: Manchester University Press, 2015).

9 Kent Fedorowich, *Unfit for Heroes: Reconstruction and Soldier Settlement in the Empire between the Wars* (Manchester: Manchester University Press, 1995). Migrating ex-servicemen could upgrade the class of travel with personal finance; War Pensions Gazette, ‘Empire Settlement’, 357.

10 G. F. Plant, *Overseas Settlement* (London: Oxford University Press, 1951), 74.

11 Despite its mammoth size, incorporating close to 9 million ex-servicemen, there has been no historical research into the organisation. The charity still functions today under the name Royal Commonwealth Ex-Services League.

12 Martin Crotty and Mark Edele, ‘Total War and Entitlement: Towards a Global History of Veteran Privilege’, *Australian Journal of Politics and History*, 2013, 59, 15; Grace Huxford *et al.*, ‘Writing Veterans History: A Conversation on the Twentieth Century’, *War & Society*, 2019, 38, 126.

13 David Gerber, ‘Introduction: Finding Disabled Veterans in History’, in David Gerber, ed., *Disabled Veterans in History* (Ann Arbor: University of Michigan Press, 2000), 20.

14 Clem Lloyd and Jacqui Rees, *The Last Shilling: A History of Repatriation in Australia* (Melbourne: Melbourne University Press, 1994); Alistair Thomson, *Anzac Memories: Living with the Legend* (Oxford: Oxford University Press, 2013); Kate Blackmore, *The Dark Pocket of Time: War, Medicine and the Australian State, 1914–1935* (Adelaide: Lythrum Press, 2008), 136.

15 Brendan Gleeson, *Geographies of Disability* (London: Routledge, 1999), 31, 67.

16 Blackmore, *The Dark Pocket of Time*, 136.

17 Peter Leese, *Shell-Shock: Traumatic Neurosis and the British Soldiers of the First World War* (London: Palgrave Macmillan, 2002), 147*–*54.

18 Fourth Annual Report of the MoP, 1920*–*1921, 24, 35.

19 Lloyd and Rees, *The Last Shilling*, 40*–*1; A. G. Butler, *Official History of the Australian Army Medical Services in the War, Volume 3* (Canberra: Australian War Memorial, 1943), 788*–*9.

20 Blackmore, *The Dark Pocket of Time*, 1.

21 ‘Repatriation Commission [hereafter RC] Outpatient Clinic, Melbourne’; Photograph taken from Harold Paynting Series, held in State Library of Victoria, Melbourne.

22 Marina Larsson, ‘Restoring the Spirit: The Rehabilitation of Disabled Soldiers in Australia after the Great War’, *Health & History*, 2004, 6, 46*–*47.

23 ‘Australia: Report on the General Administration of Pensions in the Commonwealth of Australia and on the Payment of Pensions by the RC on behalf of the MoP and matters incidental hitherto’, by G. F. Gilbert, 6 January 1926, [hereafter ‘Australia: Report’] PIN 15/1716, National Archives of England and Wales [hereafter NA].

24 Nineteenth Annual Report of the MoP, 1935*–*1936, Part Two, 34; Stephen Garton, ‘Demobilization and Empire: Empire Nationalism and Soldier Citizenship in Australia After the First World War’, *Journal of Contemporary History*, 2015, 50, 133*–*34.

25 Nineteenth Annual Report of the MoP, 1935-1936, Part Two, 5*–*6.

26 Annual Report of the RC for the year ending 30 June 1927, 7.

27 Richards, *Destination Australia*, 88.

28 War Pensions Gazette, May 1919, ‘Free Passages’, 213; August 1919, ‘Empire Settlement’, 357.

29 British Legion, *Great War Pensions* (London: British Legion, 1924), 57*–*58.

30 Out of 133,301 applications forwarded on behalf of ex-servicemen wishing to migrate to imperial territories overseas, less than one-third were accepted as suitable by overseas governments, with emphasis placed on the likelihood that a migrant would become self-reliant and employable post-passage; Plant, *Overseas Settlement*, 73.

31 Geoffrey Reaume, ‘Disability History in Canada: Present Work in the Field and Future Prospects’, *Canadian Journal of Disability Studies*, 2012, 1, 50*–*51.

32 Roe, *Australia, Britain, and Migration*, 185*–*87; Harper and Constantine, *Migration and Empire*, 58; Michael Roe, ‘‘We can die just as easy out here’: Australia and British Migration, 1916–1939’, in Stephen Constantine, ed., *Emigrants and Empire: British Settlement in the Dominions between the Wars* (Manchester: Manchester University Press, 1990), 102.

33 Roe, *Australia, Britain, and Migration*, 185; ‘Immigration – British Ex-servicemen’, A461, A349/1/6, National Archives of Australia, Canberra, 69.

34 Fedorowich, *Unfit for Heroes*, 163*–*64.

35 ‘Twelve Months’s Goal Imperial Pension’, *The Labor Daily*, 10 July 1926, 5; ‘Tragic Story of Suicide. Imperial Pensioner’s Rash Act’, *The Mercury*, 18 March 1922, 6; ‘Sunshine Conviction Upheld’, *Sunshine Advocate*, 27 September 1929, 1; ‘TB Treatment for Imperial Pensioners’, *The Sun*, 10 March 1921, 2; ‘Murder and Suicide’, *Northern Territory Times and Gazette*, 15 September 1923, 1; ‘Unhealthy Migrants: Letter to Editors’, *Evening News*, 10 November 1922, 6; ‘Letter to Editor’ *The Sydney Morning Herald*, 15 March 1922, 14; ‘British Ex-Servicemen’, *Daily Mercury*, 17 January 1923, 4.

36 C. J. Duder, ‘The Soldier Settlement Scheme of 1919 in Kenya’, (PhD Thesis, University of Aberdeen, 1978), 88.

37 Federowich, *Unfit for Heroes*, 163; Letter sent from Governor General’s Office, Melbourne, to Secretary State for the Colonies, London, 21 February 1921, PIN 15/1705, NA; BESL, First Biennial Conference Report, 1921, 10.

38 This arrangement had operated for 25 years; MoP Memo, 9 October 1919, PIN 15/1704, NA.

39 MoP Minute Sheet, October 1921, PIN 15/1705, NA.

40 Draft, the Under-Secretary of State, Colonial Office, PIN 15/1705; MoP Minute Sheet, 12 September 1922, PIN 15/1707, NA.

41 Draft, the Under-Secretary of State, Colonial Office, PIN 15/1705, NA.

42 MoP Minute Sheet, 12 September 1922, PIN 15/1707, NA.

43 MoP internal correspondence from Hore to Aitken, 18 September 1922, PIN 15/1705, NA.

44 Sixth Annual Report of the MoP, 1922*–*1923, 5.

45 MoP internal correspondence from Hore to Aitken, 18 September 1922, PIN 15/1705, NA.

46 A. P. Skerman, *Repatriation in Australia: A History of Development* (Melbourne: Repatriation Department, 1961), 15.

47 Meghan Kowalsky, ‘‘This Honourable Obligation’: the King's National Roll Scheme for Disabled Ex-Servicemen, 1915–1944’, *European Review of History*, 2007, 14, 575.

48 Skerman, *Repatriation in Australia*, 18.

49 As Carolyn Holbrook has assessed, ‘in a settler nation that is conspicuously short of unifying mythologies, the Anzac legend is uniquely powerful’; *ANZAC: The unauthorised biography* (Sydney: New South, 2014), 206; Thomson, *Anzac Memories*, 142; Fedorowich, *Unfit for Heroes*, 144*–*45; Jay Winter, *Remembering War: The Great War between Memory and Culture in the Twentieth Century* (New Haven: Yale University Press, 2006), 169*–*70; Carol Putland, ‘Tuberculosis and the Australian State: Australia’s National Anti-tuberculosis Campaign 1898–1948 An Administrative History of a Public Health Policy’, (PhD Thesis, Flinders University, 2013), 236*–*38; Marilyn Lake, ‘The Power of Anzac’, in Michael McKernan and Margaret Browne, eds, *Australia: Two Centuries of War and Peace* (Canberra: Allen & Irwin, 1998), 195.

50 Thomson, *Anzac Memories*, 126.

51 Andrew McDonald, 'The Geddes Committee and the Formulation of Public Expenditure Policy, 1921–1922’, *The Historical Journal*, 1989, 32, 643–74; Jessica Meyer, *Men of War; Masculinity and the First World War in Britain* (London: Palgrave Macmillan, 2009), 101.

52 Duder, ‘The Soldier Settlement Scheme of 1919 in Kenya’, 93.

53 The subsequent annual outlay of medical costs was subsequently reduced from £15,000,000 to £1,600,000; Nineteenth Annual Report of the MoP, 1935*–*1936, Part Two, 37; Helen Bettinson, ‘‘Lost Souls in the House of Restoration’: British Ex-Servicemen and War Disability Pensions, 1914–1930’ (PhD Thesis, University of East Anglia, 2002), 70–77, 91; Meyer, *Men of War*, 101*–*2.

54 ‘Expenditure of the MoP’, PIN 15/2601, NA.

55 Joanna Bourke, *Dismembering the male: Men’s bodies, Britain and the Great War* (London: Reaktion, 1999), 31, 54–56, 251.

56 ‘Notes on powers and policy of the MoP’, T 161/270, NA.

57 Ex-Service Welfare Society, Annual Report, 1932, 12; Ex-Service Welfare Society, Annual Report, 1936, 11, Combat Stress Archive, Surrey; BESL, Fourth Biennial Conference Report, 1927–1928, 13.

58 Bettinson, ‘Lost Souls in the House of Restoration’, 86.

59 ‘Note on Prevention of Fraud in respect of Disability Pensions’, PIN 15/789, NA.

60 Bettinson, ‘Lost Souls in the House of Restoration’, 18. For more information, see Theda Skocpol, *Protecting Soldiers and Mothers: The Political Origins of Social Policy in the United States* (Cambridge MA: Harvard University Press, 1992), 102*–*53.

61 ‘Memorandum: The Secretary, Deputy Commissioner of Pensions, RC, Melbourne, Medical Treatment Great Britain and Australia’, 15 November 1932, 1932/732, Australian National Archives, Canberra.

62
*Ibid*.

63 Commonwealth of Australia: Parliament Debates – House of Reps, Official Hansard, No. 17, 1920.

64 J. M. Shemmens, Chairman, RC, Memorandum for Auditor General, 21 July 1923, 1932/732, Australian National Archives, Canberra.

65
*Ibid*.; Annual Report of the RC for the year ending 30 June 1927, 6.

66 Annual Report of the RC for the year ending 30 June 1927, 5–6.

67 BESL, Fourth Biennial Conference Report, 1927*–*1928, 38.

68 Letter from the MoP to the Treasury, 22 August 1923, PIN 15/1673, NA.

69 Anzac Hospitals were reserved for totally incapacitated men such as paraplegics; ‘Australia: Report’, PIN 15/1716, NA.

70 Catherine Kudlick, ‘Social History of Medicine and Disability History’, in Michael Rembis, Catherine Kudlick and Kim Nielsen, eds, *The Oxford Handbook of Disability History* (Oxford: Oxford University Press, 2018), 106.

71 RC, Minute Paper, September 1931, G193 File 3, Sydney State Archive.

72 Calculated from ‘Australia: Report’, PIN 15/1716, NA.

73
*Ibid*.

74 It appears that the specialisation of Repatriation officials was dictated by population sizes; of 3,236 pensions paid to Imperial Pensioners, nearly one half were in New South Wales; *Ibid*.

75
*Ibid*.

76
*Ibid*.

77
*Ibid*.

78
*Ibid*.

79
*Ibid*.

80 Marina Larsson, ‘Families and Institutions for Shell-Shocked Soldiers in Australia after the First World War’, *Social History of Medicine*, 2009, 22, 97*–*114.

81 ‘Australia: Report’, PIN 15/1716, NA.

82 Peter Barham, *Forgotten Lunatics of the Great War* (New Haven: Yale University Press, 2004).

83 British Legion Journal, 1923, 3, 43; T. J. Mitchell and G. M. Smith, *Medical Services: Casualties and Medical Statistics of the Great War* (London: War Office, 1931), 339; Joanna Bourke, ‘The Battle of the Limbs: Amputation, Artificial Limbs and the Great War in Australia’, *Australian Historical Studies*, 1998, 29, 49*–*67. For more information on the design of the British metallic limb, see Mary Guyatt, ‘Better Legs: Artificial Limbs for British Veterans of the First World War’, *Journal of Design History*, 2001, 14, 307*–*25.

84 ‘Australia: Report’, PIN 15/1716, NA.

85 Nineteenth Annual Report of the MoP, 1935*–*1936, Part Two, 20.

86 Without a claim submitted to the Ministry in London, the department did not intervene on behalf of lunacy cases; ‘Deputation from the British Ex-Service Legion, Melbourne’, PIN 15/671; ‘Imperial Pensions: Overseas Treatment of Lunacy Cases: Instructions to Overseas Pension Authorities’, PIN 15/893, NA.

87 Annual Report of the RC for the year ending 30 June 1937, 8*–*11; Annual Report of the RC the year ending 30 June 1938, 8*–*10; Annual Report of the RC for the year ending 30 June 1939, 4*–*5.

88 This feature was recognised by both Smith and the Secretary of the Queensland Branch of the Limbless Association; ‘Visit to Brisbane’, PIN 15/671.

89 Thomson, *Anzac Memories*, 126; Lloyd and Rees, *The Last Shilling*, 244*–*46.

90 BESL, Sixth Biennial Conference Report, 1931*–*1933, 28; Skerman, *Repatriation in Australia*, 74.

91 Skerman, *Repatriation in Australia*, 74; Lloyd and Rees, *The Last Shilling*, 255; Annual Report of the RC for the year ending 30 June 1938, 3.

92 British Legion Journal, July 1929, 9, 2; April 1930, 9, 274; May 1930, 9, 304; September 1930, 10, 74; May 1931, 10, 392; January 1931, 10, 230; ‘Prematurely Aged Ex-Servicemen’, *Lancet*, 1938, 321, 215.

93 EX-SERVICE MEN (PENSIONS); HC Deb 03 June 1938 vol 336 cc2435-71; EX-SERVICE MEN; HC Deb 23 December 1937 vol 330 cc2199–259

94 British Legion Journal, April 1933, 12, 349.

95 Nineteenth Annual Report of the MoP, 1935–1936, Part Two, 10–11, 35.

96
*Ibid*., 34

97 Letter from Gilbert to C. J. Cerutty, Auditor General, Melbourne, Australia 16 May 1934; The letter was responding to prior correspondence sent from Cerutty to Gilbert on 28 December 1933; PIN 15/1716, NA.

98
*Ibid*.

99 Stephen Garton, *The Cost of War: Australian’s Return* (Oxford: Oxford University Press, 1996), 77–78; Putland, ‘Tuberculosis and the Australian State’, 221; Larsson, *Shattered Anzacs*, 97.

100 BESL, Eighth Biennial Conference Report, 1937, 19–23.

101
*Ibid*., 49–50.

102 BESL, Seventh Biennial Conference Report, 1934, 39.

103 Nineteenth Annual Report of the MoP, 1935–1936, Part Two, 37.

104
*Ibid*., 38.

105 Larsson, *Shattered Anzacs*, 215.

106 Garton, *Demobilization and Empire*, 132; Blackmore, *The Dark Pocket of Time*, 149.

107 Christopher Pierson and Matthieu Leimgruber, ‘Intellectual Roots’, in Francis Castles *et al.*, eds, *The Oxford Handbook of the Welfare State* (Oxford: Oxford University Press, 2010), 33.

108 Bettinson, ‘Lost Souls in the House of Restoration’, 72–73.

109 Smith previously worked as the Ministry official located in Ottawa, Canada. It was on relinquishing this post that he travelled to Australia and New Zealand on behalf of the Ministry.

110 Smith, ‘Memo’, 23 July 1936; Letter from Smith to the MoP, London, 1 July 1936; PIN 15/670, NA.

111 ‘Payment of Pensions in Australia, New Zealand and South Africa: Opening of an office for the payment of pensions to deal with grievances’, DO 35/246/7, NA.

112 Letter from Smith to the MoP, London, 1 July 1936, PIN 15/670; ‘Report on Visit to New South Wales State Office’, PIN 15/ 671, NA.

113 ‘Report of Visit to the Tasmanian Branch Office of the RC’; ‘Report of Proceedings of a Deputation of with Representatives of Western Australia Branch of the TB Sailors and Soldiers’ Association’; ‘Memorandum: The Deputy Commissioner, Department of Repatriation, All States: Administration on Behalf of the MoP’, PIN 15/671, NA.

114 Letter from Smith to the MoP, London, 1 July 1936, PIN 15/670, NA.

115 Smith, ‘Memo’, 2 December 1936; Letter from Smith to the MoP, London, 1 July 1936, PIN 15/670, NA

116 ‘Interview with Mr Chapman’, PIN 15/671, NA.

117 Letter from Smith to the MoP, London, 1 July 1936, PIN 15/670, NA.

118 ‘Analysis of Complaints and Grievances’, PIN 15/670; ‘Interview with Pensions Officer of the Victorian State of the RSSILA’, PIN 15/671, NA.

119 ‘Report of the Proceedings of a Deputation Representing the West Australian Branch of the Returned Sailors and Soldiers’ Imperial League of Australia, Perth’, PIN 15/671, NA.

120 In 1933, of 64,383 appeals in Britain assisted by British Legion officials, 34,202 were successful; Bettinson, ‘Lost Souls in the House of Restoration’, 270–75; Cohen, *The War Come Home*, 53.

121 ‘Form to be completed by pensioner appealing to the Pensions Appeal Tribunal’, PIN 15/675, NA.

122 Neil Diamant, *Embattled Glory: Veterans, Military Families, and the Politics of Patriotism* (Maryland: Rowman & Littlefield, 2009), 330.

123 Reid, *Broken Men*, 123; Julie Anderson, *War, Disability and Rehabilitation in Britain: ‘Soul of a Nation’* (Manchester: Manchester University Press, 2011), 48–49; Cohen, *The War Come Home*, 50.

124 Smith repeatedly asserted the Ministry in London was to remain in possession of ex-servicemen’s records; ‘Deputation Received from the New South Wales Branch of the RSSILA’; ‘Imperial Ex-Service Pensions: Memo prepared by the Returned Soldiers League’, PIN 15/671, NA.

125 ‘Visit to Canberra’, PIN 15/671, NA.

126 Smith, ‘Memo’, 2 December 1936, PIN 15/670; ‘Memorandum: Administration on Behalf of the MoP, All States’; ‘British Imperial Ex-Service Pension Council, Melbourne’; Visit to Canberra, 10–11 December, PIN 15/671, NA.

127 ‘Discussion with Officers of the Victorian State Branch of the RC’, PIN 15/671, NA.

128 ‘Report of Visit to the Tasmanian Branch Office of the RC’; ‘General Report on Visit to the Adelaide Office of the RC’, PIN 15/671; Letter from Smith to the MoP, London, 1 July 1936, PIN 15/670, NA; Nineteenth Annual Report of the MoP, 1935–1936, Part Two, 5.

129 ‘Visit to Brisbane’; ‘British Imperial Ex-Service Pension Council Received in Melbourne’; ‘Memorandum: The Deputy Commissioner, Department of Repatriation, All States, RE: Administration on behalf of the MoP’, PIN 15/671, NA.

130 Letter from Smith to the MoP, London, 1 July 1936, PIN 15/670, NA.

131 Robert Drake, *Understanding Disability Policies* (Basingstoke: Palgrave Macmillan, 1999), 128.

132 MoP, East Midlands, to MoP, London, 15 November 1921, PIN 15/56, NA; Joanna Bourke, ‘Effeminacy, Ethnicity and the End of Trauma: The Sufferings of ‘Shell-Shocked’ Men in Great Britain and Ireland’, *Journal of Contemporary History*, 2000, 35, 65.

133 Smith was relayed the same information during his time at Perth’s Repatriation Branch Office; ‘General Report on a visit to the Perth Office of the RC’, PIN 15/671, NA; Larsson, *Shattered Anzacs*, 215; Lloyd and Rees, *The Last Shilling*, 241; Annual Report of RC for the year ending 30 June 1930, 2.

134 ‘Deputation Received from New South Wales State Branch of the Returned Sailors and Soldiers League of Australia, Sydney’, PIN 15/671, NA.

135 ‘General Report on a visit made to the Perth Office of the RC’; ‘Deputation from the Representatives of the Queensland Branch of the RSSILA, Brisbane’, PIN 15/671, NA.

136 ‘Stony Broke and Walking Audio Recording: Men & women recall their most vivid memories of the Great Depression in Australia’, 1991, Sydney State Library.

137 Lake, *The Limits of Hope*, 60–61, 134; Lloyd and Rees, *The Last Shilling*, 248.

138 ‘General Report on a visit made to the Perth Office of the RC’, PIN 15/671, NA.

139 BESL, Seventh Biennial Conference Report, 1934, 28.

140 ‘They said you’d own your farm Audio Recording: Survivors of the group settlement scheme for British World War One ex-servicemen in south Western Australia describe their experiences’, 1991, Sydney State Library.

141 ‘Correspondence with the Imperial Ex-Service Association of Australia and the British Overseas Association’, LAB 2/1237/ETO130/1932, NA.

142 Richards, *Destination Australia*, 118; Roe, ‘We can just as easy die out here’, 106.

143 BESL, Seventh Biennial Conference Report, 1934, 30.

144 ‘Deputation from the Representatives of the Queensland Branch of the RSSILA, Brisbane’; ‘British Imperial Ex-Service Pension Council, Melbourne’, PIN 15/671, NA.

145 Deceased British ex-servicemen were seemingly regularly buried within the ‘Last Post’ initiative, which paid little heed to the deceased veteran’s birth-place; BESL, Seventh Biennial Report, 1934; 29–30; BESL, Eighth Biennial Report, 1937, 43–44.

146 ‘Report of the Proceedings of a Deputation Representing the West Australian Branch of the Returned Sailors and Soldiers’ Imperial League of Australia, Perth’; ‘Deputation from Representatives of the Queensland Branch of the RSSILA, Brisbane’, PIN 15/671, NA.

147 Crotty and Edele, ‘Total War and Entitlement’, 24.

148 ‘Imperial Ex-Service Pensions’, PIN 15/671, NA.

149 Letter from Smith to the MoP, London, 1 July 1936, PIN 15/670; ‘Discussion with Officers of the Victorian State Branch of the RC’, PIN 15/671, NA.

150 ‘Visit to Brisbane’, PIN 15/671, NA; Previous research has attested as many half of the AIF enlistees during the First World War were British-born migrants; Jay Winter, *Remembering the Great War*, 169; Garton, ‘Demobilization and Empire’, 130; Blackmore, *The Dark Pocket of Time*, 184–85.

151 Letter from P. E. Causwick to Repatriation Branch, G1052 Part 2, Adelaide State Archive.

152 ‘Complaints from British Ex-Servicemen’, 6 August 1935, G193 File 4, Sydney State Archive.

153 RC, Melbourne, Memorandum, 12 August 1935, G193 File 4, Sydney State Archive.

154 Letter from Smith to the MoP, London, 1 July 1936, PIN 15/670.

155
*Ibid*.

156 Letter from Smith to the MoP, London, 1 July 1936, PIN 15/670; ‘General Report on Visit to the Adelaide Office of the RC’, PIN 15/671, NA.

157 ‘Registration of Documents relating to ex-imperial Soldiers’, 11 January 1932’; G802, Adelaide Archive.

158 ‘General Report on a Visit made to the Perth Office of the RC’; ‘Visit to Brisbane’, PIN 15/671, NA.

159 ‘General Report on a visit to the Perth Office of the RC’, PIN 15/671, NA.

160 ‘British Imperial Ex-Service Pension Council, Melbourne’, PIN 15/671, NA.

161 ‘Visit to Brisbane’, PIN 15/671, NA.

162 ‘Discussion with Officers of the Victorian State Branch of the RC’, PIN 15/671, NA.

163 Previous research has described the preferential treatment of Australian ex-servicemen in employment as a ‘central plank of repatriation policy’; Thomson, *Anzac Memories*, 105, 113–14.

164 Letter from Smith to the MoP, London, 1 July 1936, PIN 15/670, NA.

165 Fedorowich, *Unfit For Heroes*, 164–65.

166 BESL, Seventh Biennial Conference Report, 1934, 29–30.

167 ‘Deputation from the BESL Received, Melbourne’; ‘Deputation from Representatives of the Western Australia Council of Ex-Service Associations’, PIN 15/671, NA.

168 Richards, *Destination Australia*, 105; Roe, ‘We can just as easy die out here’, 112; Roe, *Australia, Britain and Migration*, 156; Geoffrey Sherington, *Australia’s Immigrants: 1788–1978* (Boston: George Allen and Unwin, 1980), 112.

169 Letter from Smith to the MoP, London, 1 July 1936, PIN 15/670, NA.

170 ‘Report of the Proceedings of a Deputation Received in Perth’, PIN 15/671, NA.

171 ‘Visit to Canberra’, PIN 15/671, NA.

172 ‘British Imperial Ex-Service Council, Melbourne’, PIN 15/671, NA.

173 Just 2 years earlier, the RC described its annual workload as ‘a very heavy one’; Annual Report of the RC for the year ending 30 June 1937, 26; Annual Report of the RC for the year ending 30 June 1939, 3.

174 Letter from Gilbert to A. G. Joyce, The Treasury, Canberra, 8 August 1933, PIN 15/1716, NA.

175 The establishment and operation of the Ministry branch is discussed in ‘Cases dealt with by British MoP’, D2048 G1052 Part 2, Adelaide State Archive.

176 ‘MoP and its work’, PIN 15/2601, NA.

177 Annual Report of the RC for the year ending 30 June 1930, 2.

178 BESL, First Biennial Conference Report, 1921, 3.

179 Crotty and Edele, ‘Total War and Entitlement’, 16.

180 Francis Castles, *Comparative Public Policy: Patterns of Post-War Transformation* (Cheltenham: Edward Elgar, 1998), 93.

181 Blackmore, *The Dark Pocket of Time*, 156.

182 Thomson, *Anzac memories*, 134–35, 142.

183 David Gerber, ‘Disabled Veterans and the Wounds of War’, in Michael Rembis, Catherine Kudlick and Kim Nielsen, eds, *The Oxford Handbook of Disability History* (Oxford: Oxford University Press, 2018), 477–502.

